# Multiplex LC–MS/MS assay for simultaneous quantification of artesunate and its active metabolite dihydroartemisinin with pyronaridine, proguanil, cycloguanil, and clindamycin in pharmacokinetic studies

**DOI:** 10.1186/s12936-025-05387-6

**Published:** 2025-05-27

**Authors:** Christoph Pfaffendorf, Johannes Mischlinger, Jean Claude Dejon-Agobé, Oumou Maïga-Ascofaré, Ebenezer Ahenkan, Ayôla Akim Adegnika, Michael Ramharter, Sebastian G. Wicha

**Affiliations:** 1 Institute of Pharmacy, Department of Clinical Pharmacy, University of Hamburg, Hamburg, Germany; 2 Centre for Tropical Medicine, Bernhard Nocht Institute for Tropical Medicine & I. Department of Medicine, University Medical Center Hamburg-Eppendorf, Hamburg, Germany; 3 German Centre for Infection Research, Partner Site Hamburg-Lübeck-Borstel-Riems, Hamburg, Germany; 4 Centre de Recherches Médicales de Lambaréné, Lambaréné, Gabon; 5 Kumasi Centre for Collaborative Research in Tropical Medicine, Kumasi, Ghana; 6 Infectious Disease Epidemiology, Bernhard Nocht Institute for Tropical Medicine, Hamburg, Germany; 7 Institut Für Tropenmedizin, Eberhard-Karls-Universität Tübingen, Tübingen, Germany; 8 German Centre for Infection Research (DZIF), Tübingen, Germany; 9 Department of Pharmacology, Kwame Nkrumah University of Science and Technology, Kumasi, Ghana

## Abstract

**Background:**

Malaria, especially caused by *Plasmodium falciparum*, remains a major global health concern, particularly in sub-Saharan Africa. To combat rising drug resistance, innovative treatment approaches like triple artemisinin-based combination therapy (TACT) and multi-drug antimalarial combination therapies (MDACTs) are being explored.

**Methods:**

This study introduces a robust and validated multiplex LC–MS/MS assay for the simultaneous quantification of key antimalarial drugs and their metabolites, including artesunate, dihydroartemisinin, pyronaridine, proguanil, cycloguanil, and clindamycin. Developed in accordance with EMA guidelines, the assay ensures high accuracy, sensitivity, and stability. Serum samples were prepared through a process of protein precipitation with acetonitrile, followed by the evaporation of the supernatant. The resulting residues were then reconstituted in a 50/50 mixture of aqueous 20 mM ammonium formate buffer and methanol for the analysis.

**Results:**

The assay achieves lower limits of quantifications of 1 ng/mL for proguanil, 0.2 ng/mL for cycloguanil, 1 ng/mL for artesunate, 4 ng/mL for dihydroartemisinin, 2 ng/mL for pyronaridine, and 5 ng/mL for clindamycin. The assay was successfully applied in a pharmacokinetic study conducted as part of a clinical trial in Gabon and Ghana, assessing novel drug combinations in both children and adults against a standard of care artemisinin-based combination therapy.

**Conclusions:**

The developed assay can support the further clinical development of these TACTs and MDACTs, ultimately contributing to enhanced malaria treatment strategies.

## Background

Malaria remains a critical global health challenge, with *Plasmodium falciparum* being the primary cause of severe cases, particularly in sub-Saharan Africa [1]. Since the early 2000 s, artemisinin-based combination therapy (ACT) has been the recommended first-line treatment for uncomplicated malaria in these endemic regions [2, 3]. Initially, it was anticipated that combining multiple drugs would decelerate the development of resistance to anti-malarial drugs. However, these expectations have not come to pass. Over the past decades, resistance to both the partner drugs and partial resistance to artemisinin derivatives has emerged, originating in the Greater Mekong Subregion of Southeast Asia [1]. Alarmingly, recent reports have also identified the first cases of such partial artemisinin resistance in Africa, raising substantial concerns about its potential spread within the high-transmission areas of sub-Saharan Africa [1, 4, 5].

To effectively combat the development of specific drug resistance, one promising strategy involves implementing triple artemisinin-based combination therapy (TACT) and multi-drug anti-malarial combination therapies (MDACTs) [6, 7]. These advanced approaches aim to align the pharmacokinetic profiles of several drugs, thereby minimizing the likelihood of resistance development by ensuring that no single drug is exposed to the parasite for an extended period.

In developing these new combination therapies, it is crucial to thoroughly understand the pharmacokinetics of the drugs involved, especially concerning possible drug-drug interactions. To support this venture of developing novel anti-malarial combination therapies a robust multi-analyte assay capable of quantifying the hallmark anti-malarial drugs artesunate, its active metabolite dihydroartemisinin, pyronaridine, proguanil, its active metabolite cycloguanil, and clindamycin within a single analytical procedure was developed.

Several assays have been developed for these drugs predominantly utilizing liquid chromatography coupled with either UV detection or mass spectrometry [8–18]. One of the recently published analytical methods enables the simultaneous quantification of artesunate, dihydroartemisinin, and pyronaridine, with calibration ranges of 1–1000 ng/mL, 2–2000 ng/mL, and 1–2000 ng/mL, respectively, and a total run time of 21 min. This method encountered challenges related to the degradation of artesunate and dihydroartemisinin due to components from haemolyzed blood in plasma samples. To address these issues, an improved sample preparation procedure was proposed. Additionally, pyronaridine exhibited significant carry-over, which was resolved by extending the washout phase and including blank samples prior to the analysis of patient samples [10]. Schouten et al*.* [17] reported an assay for pyronaridine with a calibration range of 0.5–500 ng/mL and a quicker run time of 6 min, suggesting a linear gradient as a strategy to mitigate carry-over effects. Meanwhile, Pingale et al*.* [13] developed assays for the quantification of proguanil and cycloguanil, achieving calibration ranges of 1.5–150 ng/mL and 0.5–50 ng/mL, respectively, with an even shorter run time of 2.5 min. The most recent assay capable of quantifying clindamycin alongside 17 other antibiotics reached a calibration range of 0.22–21.56 µg/mL, with a run time of 6 min [18]. Notably, no current method has been published that can simultaneously analyse all potential analytes of interest.

While many single-analyte assays demonstrate shorter run times compared to multi-analyte assays and often achieve lower limits of quantification (LLOQ), multi-analyte assays present significant advantages in reducing workload during sample preparation and minimizing overall analysis time. In clinical settings, samples typically contain 2–3 analytes (excluding active metabolites), which means that relying on single-analyte assays would necessitate at least two separate sample preparation steps and analytical runs, including calibration and quality control samples. By contrast, transitioning to a multi-analyte assay allows for a single preparation step that accommodates various analyte combinations, significantly streamlining the overall analysis process.

Consequently, a multi-analyte assay with a longer run time of 16 min was developed, which, while longer than some single-analyte assays that run for 2–6 min, provides the advantage of reduced complexity and increased efficiency. Additionally, solutions proposed in previous publications to address the analytical challenges encountered were incorporated, thereby enhancing the robustness of the method. In this paper, the development, validation, and application in a pharmacokinetic study of a multiplex liquid chromatography- tandem mass spectrometry (LC–MS/MS) assay is described.

## Methods

### Chemicals and reagents

Artesunate , dihydroartemisinin , pyronaridine , proguanil , clindamycin and trimipramine-d3 (I.S.) were sourced from Sigma-Aldrich (Steinheim, Germany). Cycloguanil was procured from Hycultec GmbH (Beutelsbach, Germany). Acetonitrile, methanol, 2-propanol, dimethyl sulfoxide (DMSO), ammonium formate, formic acid and drug free human serum were also procured from Sigma-Aldrich (Steinheim, Germany) and were LC–MS Grade. Blank Human EDTA-Plasma was obtained from the patients of the MultiMal Study at the Centre de Recherches Médicales de Lambaréné (CERMEL) (Lambaréné, Gabon) and the Kumasi Centre for Collaborative Research (KCCR) site at the St. Francis Xavier Hospital (Assin Foso, Ghana).

### Equipment

The method was developed on a 1290 Infinity high performance liquid chromatography (HPLC) II (Agilent Technologies, California, USA) coupled to a QTRAP 5500 (SCIEX, Framingham, Massachusetts, USA) electrospray ionization mass spectrometer. A reverse-phased mixed-mode column (Atlantis Premier BEH C18 AX VanGuard FIT, 150 mm × 2.1 mm, particle size 2.5 µm) from Waters Corporation (Milford, Massachusetts, USA) was used to separate the analytes.

### Liquid chromatography-tandem mass spectrometry conditions

Two eluents were used for the separation of the analytes. Eluent A consisted of 90% 10 mM aqueous ammonium formate buffer, 10% acetonitrile, and 0.025% formic acid. Eluent B consisted of acetonitrile containing 0.1% formic acid. The HPLC method used to separate the analytes is shown in Table 1.

**Table 1:** Parameters of the chromatographic  method

Time (min)	Flow rate (mL/min)	Eluent A	Eluent B
0	0.3	100	0
2.5	0.3	100	0
6	0.3	65	35
7	0.3	40	60
10	0.3	40	60
11	0.3	0	100
13.5	0.3	0	100
14	0.4	100	0
16	0.4	100	0

**Fig. 1:**
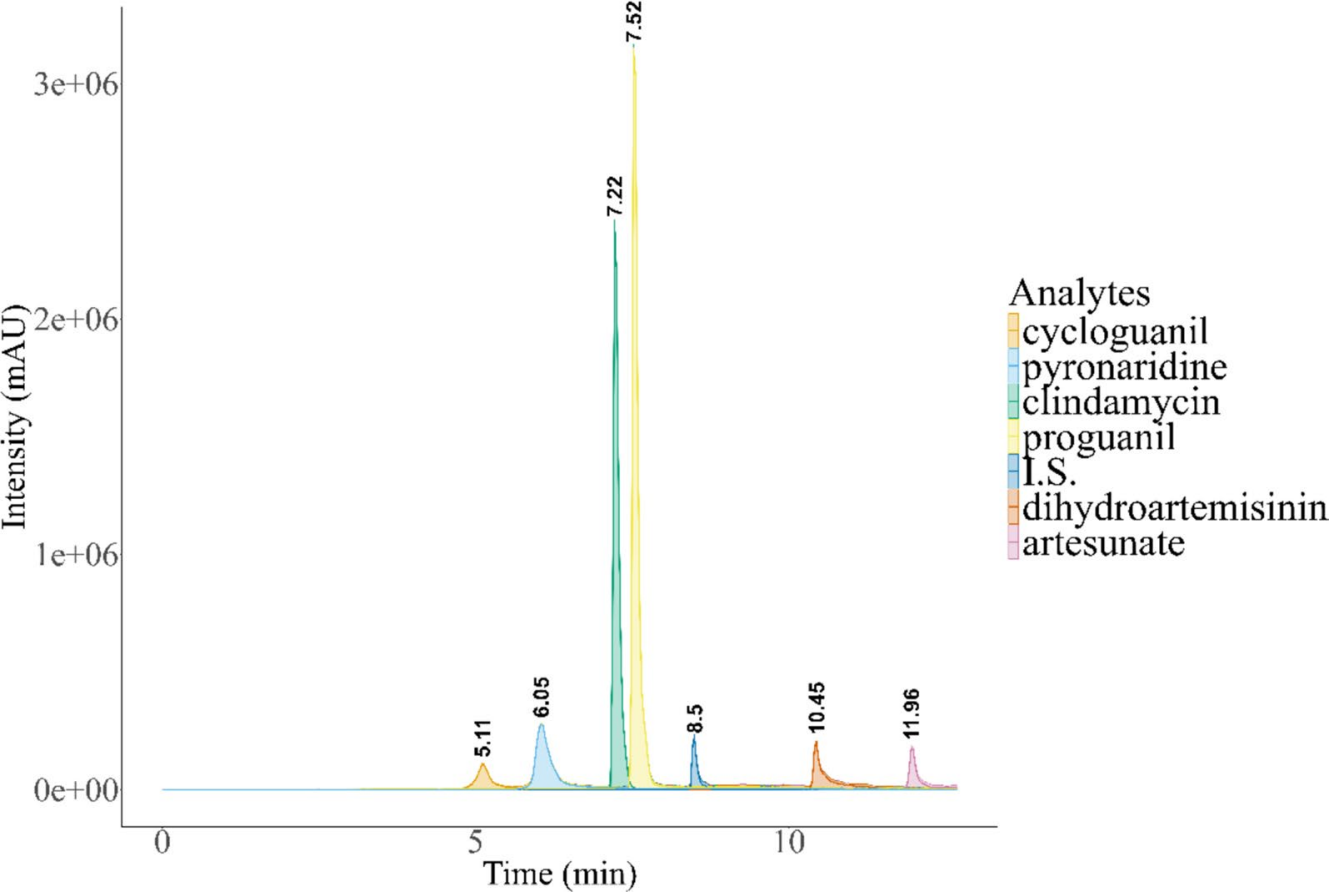
Representative chromatogram of a QCM sample showing each analyte and its respective retention time. The chromatogram is constructed from the extracted product ion intensities of each analyte

Direct injection of a reference solution of each analyte was used to find the optimal parent ion and fragment, as well as the optimal parameters. The fragment with the strongest signal was chosen as the quantifier and the second strongest as the qualifier. The chosen fragments and the parameters are shown in Table 2. To ensure measured peaks were coming from the analyte, the ion ration of each measured sample was tested. An automatic flow injection analysis was used to find the optimal source parameters for the analyte mixture. At the end of the run, a cleaning phase was added where the source was switched into negative mode. This was necessary as the signal intensity dropped for subsequent runs when this phase was not included. The final source parameters are shown in Table 3. A representative chromatogram of the separation of the analytes is shown in Fig. 1.

**Table 2:** Fragments and parameters used for each analyte and the internal standard (IS)

	Precursor Ion	Product Ion (Quantifier)	Product Ion (Qualifier)	Dwell Time (msec)	Declustering Potential (volts)	Entrance Potential (volts)	Collision Energy (volts)	Collision Cell Exit Potential (volts)
pyronaridine	518.0	447.0	298.1	150	146	10	31	40
clindamycin	425.1	126.1	70.1	150	146	10	35	12
proguanil	254.1	170.2	111.0	150	96	10	27	14
cycloguanil	252.1	195.1	153.1	150	76	10	27	14
artesunate	402.2	267.3	107.1	150	56	10	15	10
dihydroartemisinin	302.15	163.2	77.0	150	61	10	23	14
trimipramine-D3 (IS)	298.2	103.2	–	150	101	10	23	12

Each parent ion is [M + H]^+^ except for artesunate and dihydroartemisinin which are [M + NH4]^+^

**Table 3:** Source parameters of the method

	Curtain Gas (psi)	Collision Gas	IonSpray Voltage (volt)	Temperature (°C)	Nebulizer Gas (psi)	Heater Gas (psi)
Phase 1 (0–12.7 min)	20	Medium	5000	500	30	70
Phase 2 (12.7–16.0 min)	20	Medium	− 4500.0	500	30	70

The method is split into 2 phases. The measurement phase 1 and a cleaning phase 2 where the source is switched to negative mode

### Standard solutions

#### Internal standard

The choice of trimipramine-d3 as the internal standard was based on a previous publication which found it applicable for artemisinin derivatives as well as partner drugs like pyronaridine [10]. Thus, it was tested and found to be a suitable internal standard for this assay as well. A 5 ng/mL trimipramine-d3 solution in MeOH was used to spike the samples.

#### Stock solutions

Individual stock solutions for each analyte were prepared at 5 mg/mL in DMSO, except for cycloguanil which was prepared at 1 mg/mL in DMSO. A mixed stock of all analytes was prepared (artesunate: 50 µg/mL, dihydroartemisinin: 200 µg/mL, pyronaridine: 100 µg/mL, proguanil: 50 µg/mL, cycloguanil: 10 µg/mL, clindamycin: 250 µg/mL) in DMSO and used to prepare 8 calibration standards (CS) and 6 quality controls (QC) levels (Table 4). Different stocks were used for CS and QC. The mixed analyte stock was stored at − 80 °C and stocks to prepare CS and QC were prepared freshly on the day CS and QC samples were prepared.

**Table 4:** Calibration range and concentrations levels of the QC samples for each analyte

Analyte	QCLLOQ (ng/mL)	QCL (ng/mL)	QCM (ng/mL)	QCH (ng/mL)	Dilution method A		Dilution method B		Calibration Range (ng/mL)
					QCD (ng/mL)	QCD2 (ng/mL)	QCD (ng/mL)	QCD2 (ng/mL)	
Pyronaridine	2	6	100	170	4000	2000	3333	1666	2–200
Clindamycin	5	15	250	425	18,000	9000	8333	4166	5–500
Proguanil	1	3	50	85	2000	1000	1666	833	1–100
Cycloguanil	0.2	0.6	10	17	400	200	333	166	0.2–20
Artesunate	1	3	50	85	2000	1000	1666	833	1–100
Dihydroartemisinin	4	12	200	340	8000	4000	6666	333	4–400

QCLLOQ is equal to the lowest calibration standard, QCL is three times the concentration of QCLLOQ, QCM which is 50% of the highest calibration standard, QCH is 85% of the highest calibration standard, QCD is 20 × the maximum calibration standard (36 × for clindamycin) for method A and approximately 20 × QCH for method B and QCD2 is 10 × the maximum calibration standard (18 × for clindamycin) for method A and approximately 10 × QCH for method B.

### Sample preparation

To prepare the CS and QCs, standard solutions prepared from the mixed stock were used to spike serum in a 1:50 ratio. 50 µL of the ice-cold I.S. solution was added to 100 µL of the sample and vortexed for 5 s. Afterwards, 900 µL ice-cold acetonitrile was added to the samples and vortexed for 10 min. Thereafter, the samples were incubated on ice for 10 min and then vortexed again for 10 s. After centrifuging the samples for 10 min at 17,968 G, 950 µL of the supernatant was put in a new tube and evaporated to complete dryness with pressurized air at room temperature. The samples were then reconstituted with 300 µL of a reconstitution solution (50:50 20 mM ammonium formate (pH 4) and methanol) and vortexed for 20 s. Subsequently, the samples were centrifuged again for 10 min at 17,968 G and 150 µL of the supernatant was transferred to an HPLC-Vial.

### Bioanalytical method validation

The method was validated based on the European Medicines Agencies (EMA) Guideline for bioanalytical method validation [19]. All results were obtained from the peak areas of the extracted ion chromatograms of the quantifier ion corresponding to each analyte.

#### Selectivity and carry-over

In order to assess the selectivity of the method, 6 human serum samples and six EDTA-plasma samples (blanks), as well as samples without the internal standard (double blank) were measured. Results of samples spiked with the analytes at the lowest calibration level were compared with these results. Blank sample signals of the analytes must be below 20% of the lowest calibrator's signal, and the double blank signal under 5% of the internal standard's signal. The carry-over was tested by injecting a solvent sample after the highest calibrator and comparing its signals with those of the lowest calibrator.

#### Linearity and LLOQ

The linearity of the calibration curves, covering the concentrations shown in Table 4, was assessed over three runs. In each run, eight concentration levels were evaluated in duplicate for each analyte. A linear least squares regression model weighted by 1/x^2^ was used to create calibration curves by plotting the area ratios (analyte to I.S.) against the nominal concentrations. The lowest concentration on the calibration curve that could still be measured with acceptable accuracy and precision was defined as the LLOQ.

#### Accuracy and precision

Three runs were conducted on 3 different days to evaluate accuracy and precision. Therefore, four concentration levels of QC samples with five replicates each were tested. The intra-day accuracy and precision were evaluated by assessing each validation run separately, and the inter-day accuracy and precision by pooling all three validation runs. In order to be deemed accurate, the concentrations of the samples should not have deviated more than 15% from the nominal concentration, with the exception of the LLOQ, which ought to be within 20%. The degree of precision was acceptable if the coefficient of variation (CV) did not exceed 15% or 20% for the LLOQ.

#### Dilution integrity

To ensure that all clinical samples could be analysed, two dilutions methods were tested. With method A, 20-fold and 40-fold dilutions were achieved through a two-step process. First, the sample was diluted 1:4 using the reconstitution solution following the sample preparation. Second, the injection volume was reduced to decrease the signal intensity. To achieve a total dilution of 1:20, 2 µL of the sample was injected instead of the standard 10 µL. Similarly, to achieve a 1:40 dilution, 1 µL was injected instead of 10 µL. Two QC levels were prepared to evaluate the dilution integrity. The first level involved a sample with a concentration equivalent to 20 times the highest calibrator (QCD), used to test the 40-fold dilution. The second level involved a sample with a concentration equivalent to 10 times the highest calibrator (QCD2), used to test the 20-fold dilution. For clindamycin, the concentrations were adjusted to 36-fold and 18-fold relative to the highest calibrator. The samples were prepared and analysed across three separate accuracy and precision runs, with each run conducted with 5 replicates to ensure reliability and reproducibility. For method B, 10-fold and 20-fold dilutions were achieved by diluting samples with serum before the sample preparation. The dilution integrity was assessed by testing 5 replicates for both 10-fold and 20-fold dilutions. The dilution integrity was deemed as acceptable when the measured concentration did not deviate from the nominal concentration by more than 15% and the CV did not exceed 15%.

#### Stability

For stability tests, five replicates were used for each of the low- and high-QC levels. Stability was considered to be proven when the measured concentration did not deviate from the nominal concentration by more than 15% and the CV was below 15%. Times for the stability testing were chosen based on the maximal time the samples were expected to be stored at these conditions. For the bench top stability test, samples were kept at room temperature for 90 minutes before analysis. For refrigerator stability, samples were kept at 4 °C for 12 h before being analysed. The freeze–thaw stability test involved freezing samples at − 80 °C, then thawing and refreezing them for at least 12 h, repeating this cycle three times. Analyses were conducted immediately after the third thaw. For autosampler stability, samples (after extraction) were stored in the autosampler for 45 h at 10 °C. Long-Term stability was measured after storing samples at − 80 °C for 1 year. Both low and high concentration levels were measured in triplicate as a baseline before storage and after 7 months and 1 year to calculate the recovery of each analyte.

#### Stock stability

To evaluate the stability of the analytes in the mixed stock solution, it was stored at −80 °C for five months. Samples at the same concentration were prepared from both the freshly prepared stock and the stored stock for comparison. Three replicates were measured from two different batches of the mixed stock solution for both the fresh and stored samples to ensure consistency and reliability in the analysis.

#### Matrix and anticoagulant effect

The matrix effect was evaluated by spiking blank plasma samples from six individuals with the analytes after sample preparation and comparing their peak areas, normalized by the I.S., to those obtained without the plasma matrix. Furthermore, the influence of the anticoagulant EDTA was examined by comparing these peak areas to those from samples prepared with human serum. For each sample, the ratios of peak area were calculated, and the CV was utilized to evaluate the variability. In order to guarantee minimal interference from the matrix, it is recommended that the variability not exceed 15% CV.

### Pharmacokinetic application

The assay was developed to analyse the pharmacokinetic samples of the MultiMal trial, a phase II proof of concept trial testing novel combination therapies against uncomplicated *Plasmodium falciparum* infections. The study was conducted at two locations. At CERMEL in Lambaréné Gabon and KCCR/St. Francis Xavier Hospital in Assin Foso, Ghana. The clinical trial was approved by the relevant Independent Ethics Committees, national Institutional Review Boards, and local regulatory authorities. The study protocol was registered online prior to start of recruitment into this clinical trial (pactr.samrc.ac.za, PACTR202008909968293). All patients taking part in the study gave informed consent.

Samples after administration of 150 mg artesunate (dihydroartemisinin), 540 mg pyronaridine-tetraphosphate, 200 mg proguanil (cycloguanil) and 150 mg clindamycin were taken including the following time points: 0, 0.25, 0.5, 0.75, 1.5, 3, 5, 8, 24, 48 h, day 7, day 14, day 21, day 28, day 35, and day 42. Blood samples were collected in 2 mL EDTA tubes and subsequently centrifuged at 1300*g* for 10 min at room temperature to separate the plasma. The resulting plasma was carefully transferred into polypropylene tubes. To preserve sample integrity, the tubes were then stored at − 80 °C until the time of analysis. The samples were transported from Lambaréné and Assin Foso to Hamburg for the LC–MS/MS analysis. The transport was done on dry ice with constant temperature tracing. In this application, only one representative patient per analyte is shown, as the full data will be published in a separate publication.

## Results

### Liquid chromatography-tandem mass spectrometry conditions

The search for a suitable column that ensured optimal retention and peak shape for all analytes proved to be a challenge due to the vastly different chemical properties of the compounds involved. Multiple column types, including C18, Shielded-C18, and Fluoro-phenyl columns were tested. The initial aim was to incorporate fosmidomycin into the assay, which prompted the evaluation of a mixed-mode column designed for the retention of highly polar and acidic compounds. Although the inclusion of fosmidomycin in the assay did not ultimately succeed, it was found that the mixed-mode column provided the best peak shapes and sufficient separation from the matrix. The aim was to minimize the run time as much as possible, while retaining enough separation from the dead volume at about 2 min to avoid interferences with matrix components. However, increasing the initial concentration of acetonitrile to shift the peaks closer to the 2-min mark compromised the retention and peak shapes of cycloguanil and pyronaridine. Additionally, a cleaning phase using 100% acetonitrile, followed by a 2-min equilibration phase, was necessary at the end of each run to mitigate some of the carry-over of pyronaridine and ensure consistent retention times. To further reduce the run time, step-wise gradient changes were added wherever feasible. This adjustment successfully reduced the overall run time from over 20 min to 16 min. Ultimately, a method that featured a step-wise gradient with a small linear gradient applied at the beginning was developed. This method utilized ammonium formate as a buffer in the aqueous phase, combined with the addition of 0.1% formic acid to the acetonitrile. This optimization yielded the best peak shapes while also achieving the shortest run time. Furthermore, a problem with carry-over of pyronaridine was observed, which was slightly improved with a wash-out phase at the end of the run.

### Bioanalytical method validation

The method successfully validated according to the EMA Guideline for Bioanalytical Method Validation. The results of the validation are presented below.

#### Selectivity and carry-over

**Fig. 2:**
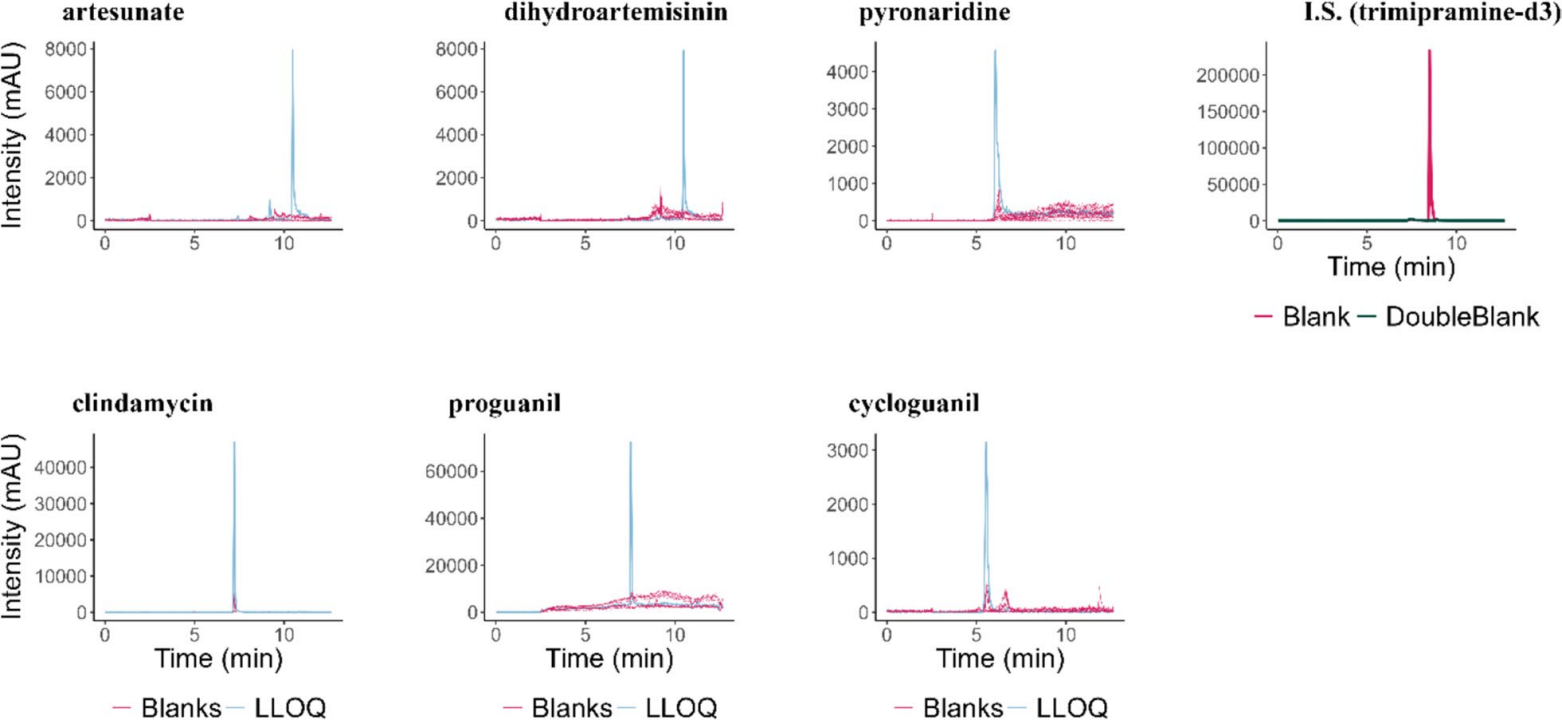
Chromatograms of 6 blank serum samples, 6 blank plasma samples, and a LLOQ sample are shown for each analyte. A representative chromatogram of a blank matrix sample, and a double blank matrix sample are shown for the internal standard

Selectivity was successfully demonstrated for all analytes, as blank samples showed no peaks exceeding 20% of the LLOQ area for any analyte, as depicted in Fig. 2. However, carry-over was observed for pyronaridine and dihydroartemisinin, each exhibiting signals greater than 20% of the LLOQ in a solvent injection following the highest calibrator, as detailed in Table 5. To address this issue, the analysis was structured with non-blinded samples, sorted by their expected concentrations. To minimize carry-over effects, three solvent injections were performed after samples with high concentrations to ensure that subsequent analyses of samples with low concentrations were not distorted.

**Table 5:** Carry-over of each analyte after the highest calibrator

Analyte	Area of LLOQ in solvent injection after highest calibrator (%)
Artesunate	12.8
Dihydroartemisinin	27.3
Pyronaridine	79.6
Clindamycin	1.5
Proguanil	6.8
Cycloguanil	2.8

Values are given as the area percentage of the LLOQ signal area

#### Linearity and LLOQ

The peak area ratios of the analytes to the internal standard demonstrated proportionality to the concentration for all analytes. The calibration curves showed linearity and were fitted using least squares regression, weighted by 1/x^2^. Each calibration curve exhibited correlation coefficients exceeding 0.99 for every analyte across all accuracy and precision runs. The deviations of the back-calculated values from the nominal standard concentrations were within 15%, except at the LLOQ, where deviations were maintained at less than 20%.

#### Accuracy and precision

All analytes exhibited deviations of less than 15% from the nominal concentration across all quality control levels, except at the LLOQ, where deviations were within 20%. This level of accuracy was consistently achieved both in individual runs and when samples from all three runs were pooled together. Precision was also confirmed, with the CV being less than 15% for all analytes across all concentration levels and runs and less than 20% at the LLOQ. The complete results from the accuracy and precision runs are detailed in Table 6.

**Table 6:** Results of the precision and accuracy runs

		Nominal concentration (ng/mL)	Day 1			Day 2			Day 3			Interday		
			Calculated concentration (ng/mL)	Accuracy (%)	Precision (%CV)	Calculated concentration (ng/mL)	Accuracy (%)	Precision (%CV)	Calculated concentration (ng/mL)	Accuracy (%)	Precision (%CV)	Calculated concentration (ng/mL)	Accuracy (%)	Precision (%CV)
Pyronaridine	QCLLOQ	2.0	1.90	94.9	6.4	1.77	88.5	2.5	1.81	90.5	5.5	1.82	91.0	5.6
	QCL	6.0	5.52	92.0	3.9	5.60	93.3	5.3	5.19	86.5	12.5	5.45	90.9	8.6
	QCM	100.0	98.40	98.4	4.9	87.80	87.8	12.4	100.60	100.6	5.3	96.20	96.2	10.0
	QCH	170.0	178.67	105.1	1.9	168.64	99.2	3.6	184.79	108.7	2.5	177.48	104.4	4.6
Clindamycin	QCLLOQ	5.0	4.23	84.6	3.5	4.59	91.7	3.6	4.82	96.4	3.1	4.54	90.8	6.4
	QCL	15.0	14.24	94.9	2.5	14.48	96.5	3.5	15.20	101.3	3.2	14.69	97.9	3.9
	QCM	250.0	245.00	98.0	2.5	213.25	85.3	2.1	250.25	100.1	4.1	237.00	94.8	7.7
	QCH	425.0	423.73	99.7	2.6	389.73	91.7	4.7	434.78	102.3	2.1	415.23	97.7	5.6
Proguanil	QCLLOQ	1.0	0.91	91.2	4.5	0.85	84.5	3.4	0.96	96.2	2.4	0.90	90.2	6.4
	QCL	3.0	2.81	93.6	1.3	2.77	92.4	4.6	2.92	97.3	2.6	2.84	94.6	3.8
	QCM	50.0	46.65	93.3	3.1	42.85	85.7	0.6	49.25	98.5	3.6	46.25	92.5	7.0
	QCH	85.0	82.54	97.1	1.9	74.80	88.0	4.5	86.11	101.3	4.4	81.18	95.5	6.9
Cycloguanil	QCLLOQ	0.2	0.17	85.9	8.9	0.19	97.0	5.2	0.18	91.6	4.0	0.18	91.1	8.2
	QCL	0.6	0.54	89.6	6.6	0.57	95.6	4.3	0.55	91.9	4.0	0.55	92.4	5.5
	QCM	10.0	8.65	86.5	2.6	8.94	89.4	2.4	9.77	97.7	2.9	9.14	91.4	5.6
	QCH	17.0	15.93	93.7	5.9	15.52	91.3	5.6	17.17	101.0	2.1	16.22	95.4	6.4
Artesunate	QCLLOQ	1.0	1.00	100.3	2.2	0.88	87.7	5.0	0.93	92.8	6.0	0.94	93.5	7.1
	QCL	3.0	3.02	100.6	4.8	2.75	91.8	1.4	2.97	99.1	3.1	2.93	97.7	5.6
	QCM	50.0	48.90	97.8	4.1	43.90	87.8	1.2	50.10	100.2	4.8	47.95	95.9	7.1
	QCH	85.0	87.04	102.4	1.7	75.74	89.1	3.8	87.81	103.3	2.7	83.39	98.1	7.1
Dihydroartemisinin	QCLLOQ	4.0	3.75	93.8	6.8	3.79	94.7	11.4	3.71	92.7	5.5	3.74	93.5	8.4
	QCL	12.0	11.14	92.8	3.2	11.42	95.2	6.2	11.48	95.7	3.5	11.33	94.4	4.8
	QCM	200.0	195.80	97.9	4.3	185.60	92.8	4.3	189.00	94.5	5.5	190.60	95.3	5.4
	QCH	340.0	339.66	99.9	1.3	333.54	98.1	2.6	328.44	96.6	2.3	333.88	98.2	2.6

For each analyte and quality control level five replicates were measured

#### Dilution integrity

The dilution integrity was successfully demonstrated for both method A and method B for all analytes for method A, samples were pooled across all three validation runs to evaluate both accuracy and precision. The results showed that both methods dilutions had deviations from the nominal concentration of less than 15%, with a CV also below 15%. Complete results are presented in Table 7.

**Table 7:** Results of the dilution integrity tests

Analyte	QCD		QCD2	
	Accuracy (%)	Precision (%CV)	Accuracy (%)	Precision (%CV)
Method A				
Pyronaridine (n = 15)	93.9	7.9	92.3	5.8
Clindamycin (n = 15)	86.0	6.4	85.4	6.4
Proguanil (n = 15)	98.7	6.0	101.2	7.0
Cycloguanil (n = 15)	104.1	6.6	108.4	5.5
Artesunate (n = 15)	105.6	6.5	106.5	5.7
Dihydroartemisinin (n = 15)	91.5	5.3	91.1	7.1
Method B				
Pyronaridine (n = 5)	92.8	7.0	103.1	6.8
Clindamycin (n = 5)	91.8	5.5	97.4	4.9
Proguanil (n = 5)	96.0	4.8	105.2	5.3
Cycloguanil (n = 5)	95.8	3.4	106.8	6.5
Artesunate (n = 5)	92.4	6.2	96.8	5.8
Dihydroartemisinin (n = 5)	90.2	6.7	93.5	6.5

For method A, QCD is 20 × the maximum calibration standard to test 40-fold dilution and QCD2 is 10 × the maximum calibration standard to test 20-fold dilution (and 36 × and 18 × the highest calibration standard for clindamycin)

For method B, QCD is 20 × the QCH to test 20-fold dilution and QCD2 is 10 × QCH to test 10-fold dilution

#### Stability

The stability of all analytes in the chosen testing condition could be shown. No degradation was observed, and all samples remained within 15% of the nominal concentration. Results are presented in Table 8.

**Table 8:** Stability results for each analyte for storage at room temperature (RT) for 1.5 h, in the fridge (FG) at 4 °C for 12 h, autosampler (AS) at 10 °C for 45 h, and freeze–thaw (FT) from − 80 °C for 3 cycles

		Nominal concentration (ng/mL)	RT			FG			AS			FT		
			Calculated concentration (ng/mL)	Accuracy (%)	Precision (%CV)	Calculated concentration (ng/mL)	Accuracy (%)	Precision (%CV)	Calculated concentration (ng/mL)	Accuracy (%)	Precision (%CV)	Calculated concentration (ng/mL)	Accuracy (%)	Precision (%CV)
Pyronaridine	QCL	6.0	5.80	96.7	11.8	5.90	94.7	11.8	6.01	100.2	5.7	6.34	105.7	4.2
	QCH	170.0	184.11	108.3	3.5	168.3	99.0	4.0	180.71	106.3	1.2	183.77	108.1	1.2
Clindamycin	QCL	15.0	14.60	97.3	3.50	15.62	104.10	2.60	13.64	90.9	1.50	15.11	100.7	3.90
	QCH	425.0	404.18	95.1	7.30	408.43	96.1	2.60	387.18	91.1	2.20	434.78	102.3	9.30
Proguanil	QCL	3.0	3.13	104.3	1.60	2.89	96.2	4.50	2.94	98.1	1.10	3.09	102.9	2.80
	QCH	85.0	84.83	99.8	3.50	83.30	98.0	3.70	80.16	94.3	1.50	81.01	105.3	1.30
Cycloguanil	QCL	0.6	0.54	90.1	7.00	0.54	90.6	8.00	0.56	92.5	12.30	0.56	93.5	3.10
	QCH	17.0	18.46	108.6	2.50	17.56	103.3	3.20	16.42	96.6	1.50	17.32	101.9	0.90
Artesunate	QCL	3.0	3.19	106.3	5.60	2.95	98.2	4.80	2.94	98.1	1.10	3.34	111.2	4.20
	QCH	85.0	91.46	107.6	2.20	85.85	101.0	2.80	80.16	94.3	1.50	89.17	104.9	2.00
Dihydroartemisinin	QCL	12.0	12.84	107.00	2.80	11.74	97.8	3.80	11.05	92.1	9.20	13.34	111.2	3.10
	QCH	340.0	352.92	103.80	2.00	331.84	97.6	2.80	359.04	105.6	0.30	346.46	101.9	2.30

#### Long-term stability

Long-term stability results are shown in Table 9. Except for clindamycin and proguanil all analytes showed recoveries above 85% at 7 months. Proguanil however showed a recovery of above 85% at 12 months. At 12-month, dihydroartemisinin also dropped below an 85% recovery.

**Table 9:** Long-term stability after 7 and 12 months at −80 °C

Analyte	7 months	12 months
	Recovery (%)	Recovery (%)
Pyronaridine	96.9 (6.0%CV)	108.8 (1.5%CV)
Clindamycin	76.5 (2.0%CV)	78.0 (3.8%CV)
Proguanil	82.8 (3.3%CV)	95.0 (1.3%CV)
Cycloguanil	102.7 (4.0%CV)	97.3 (3.9%CV)
Artesunate	93.0 (6.3%CV)	86.4 (1.2%CV)
Dihydroartemisinin	88.5 (6.5%CV)	80.6 (3.5%CV)

QCM was measured in triplicate at each time point. Results are shown as recovery in % compared to day 0

#### Stock stability

No significant degradation of the analytes was observed after five months of storage at − 80 °C, with all analytes remaining within 15% of their nominal concentrations. Therefore, it was concluded that the mixed stock solution can be used for up to five months after preparation when stored at − 80 °C. Complete results are presented in Table 10.

**Table 10:** Recovery of each analyte from a mixed stock containing all analytes stored at − 80 °C for 5 months compared to a newly prepared stock

Analyte	Recovery old Stock (%)
Pyronaridine	95.1
Clindamycin	94.0
Proguanil	95.2
Cycloguanil	88.5
Artesunate	95.2
Dihydroartemisinin	94.8

#### Matrix and anticoagulant effect

The tested matrix effect demonstrated a CV of less than 15% for all analytes, thereby successfully passing the test. Additionally, the effects of plasma compared to serum, including the impact of the anticoagulant, were evaluated. All analytes exhibited an effect close to one, indicating minimal differences in the matrix effect between serum and plasma. The results of the matrix effect and the anticoagulant effect are detailed in Tables 11 and 12, respectively.

**Table 11:** Matrix effect of all analytes

Analyte	QCL		QCH	
	Matrix effect	Precision (%CV)	Matrix effect	Precision (%CV)
Pyronaridine	1.31	6.1	1.35	5.4
Clindamycin	1.35	6.1	1.38	6.3
Proguanil	1.11	8.0	1.11	6.0
Cycloguanil	1.20	6.8	1.10	5.7
Artesunate	1.19	5.8	1.13	6.5
Dihydroartemisinin	1.43	3.9	1.46	3.9

Blank samples of six study participants were spiked after sample preparation and compared to a sample without matrix

Matrix effect was calculated as the ratio of the area of the analyte with and without matrix (area normalized over the internal standard)

**Table 12:** Anticoagulant effect on all analytes

Analyte	QCL		QCH	
	Anticoagulant effect	Precision (%CV)	Anticoagulant effect	Precision (%CV)
Pyronaridine	0.98	6.1	1.06	5.4
Clindamycin	0.93	6.1	0.94	6.3
Proguanil	1.04	8.0	1.03	6.0
Cycloguanil	1.07	6.8	1.01	5.7
Artesunate	1.06	5.8	1.06	6.5
Dihydroartemisinin	1.12	3.9	1.15	3.9

Blank EDTA plasma of six study participants was spiked after sample preparation and compared to a sample without anticoagulant (Human Serum)

Anticoagulant effect was calculated as the ratio of the area of the analyte in matrix with anticoagulant and matrix without anticoagulant (area normalized over the internal standard)

### Pharmacokinetic application

**Fig. 3:**
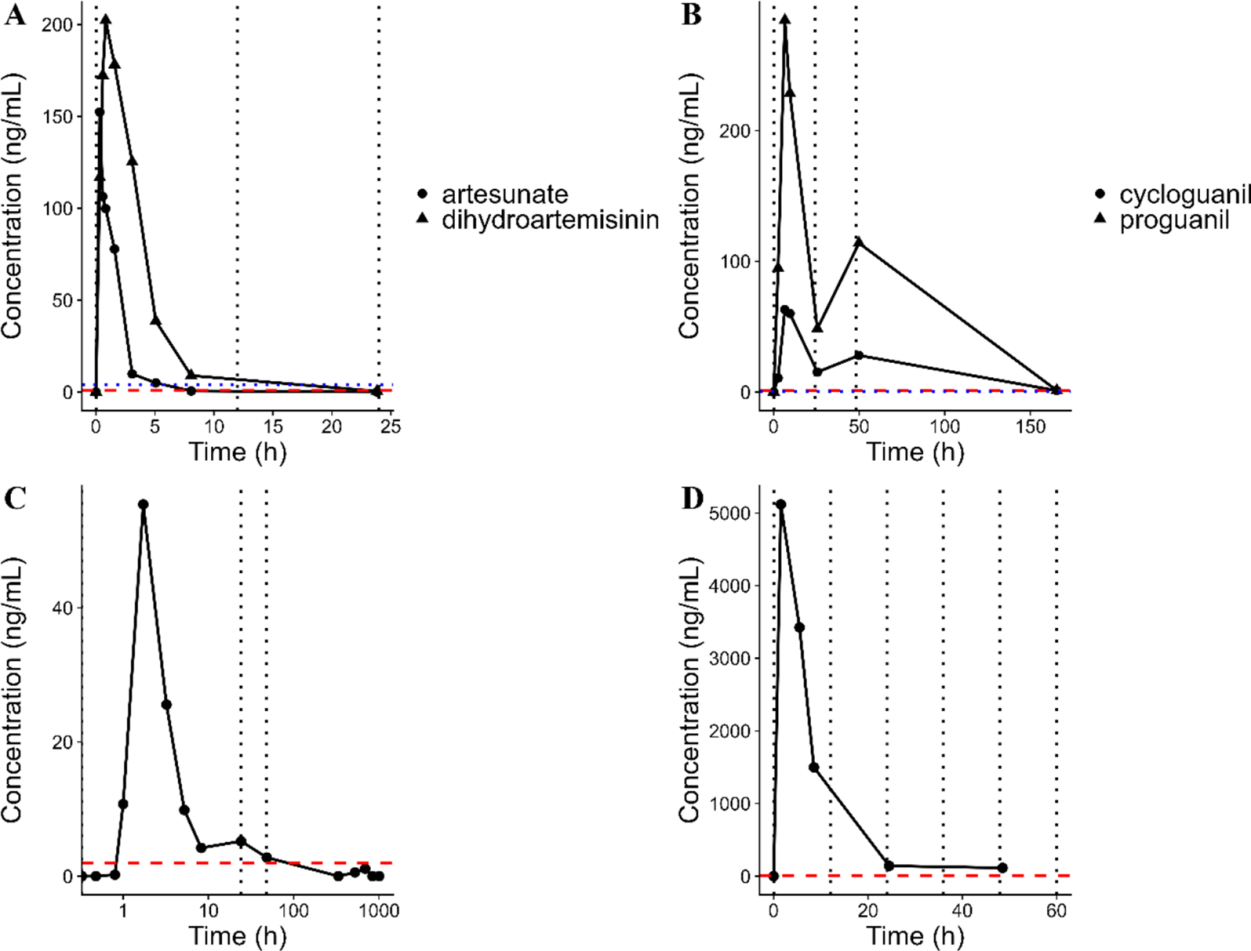
**A** Concentration–time profile of one representative patient for artesunate and dihydroartemisinin. The dashed red line represents the LLOQ of artesunate (1 ng/mL) and the dotted blue line indicates the LLOQ of dihydroartemisinin (4 ng/mL). The dotted horizontal black line shows the artesunate doses. **B** Concentration–time profile of one representative patient for proguanil and cycloguanil. The dashed red line represents the LLOQ of proguanil (1 ng/mL) and the dotted blue line the LLOQ of cycloguanil (0.2 ng/mL). The dotted horizontal black line represents the proguanil doses. **C** Concentration time profile of one representative patient for pyronaridine. The dotted red line represents the LLOQ of pyronaridine (2 ng/mL). The dotted horizontal black line shows the pyronaridine doses. **D** Concentration–time profile of one representative patient for clindamycin. The dashed red line represents the LLOQ of clindamycin (5 ng/mL). The dotted horizontal black line shows the clindamycin doses. Samples at later time points which are not shown were excluded if no quantifiable peak was observed

The pharmacokinetic study was successfully conducted, yielding detailed concentration–time profiles for all targeted analytes. Figure 3 illustrates the concentration–time profiles for all 4 drugs and their respective active metabolites.

The pharmacokinetic results exhibited similarities to those reported in the literature. The observed maximal concentration (Cmax) values of approximately 150 ng/mL for artesunate and 200 ng/mL for dihydroartemisinin, both of which demonstrated rapid elimination. These anti-malarial drugs are known for their considerable variability in pharmacokinetics, with reported mean Cmax values ranging from 49 to 451 ng/mL for artesunate and from 228 to 2034 ng/mL for dihydroartemisinin [20]. While the example patient highlighted in this study represents the lower end of the Cmax spectrum for dihydroartemisinin, most patients typically achieve higher Cmax values, consistent with literature. Regarding pyronaridine, literature data in plasma are limited, with reported Cmax values ranging between 76.2 and 495.8 ng/mL [21–23]. The only study involving more than one patient (n = 5) by Ramanathan et al*.* [23] reported a Cmax of 120 ± 30 ng/mL in individuals with acute uncomplicated falciparum malaria after completing a 3-day regimen of 700 mg of pyronaridine. These findings suggest that pyronaridine’s pharmacokinetics exhibit significant variability. In this study, a Cmax of 55 ng/mL was recorded. It is important to note that the calculated Cmax corresponds to the first dosing interval, while that of Ramanathan et al*.* [23] was observed after the third dose. This value aligns with the reported ranges, particularly considering the lower dosage administered to the patients and the fact that pyronaridine may accumulate due to its long half-life. Additionally, a previous study examining proguanil in children with acute falciparum malaria reported mean Cmax values of 244 ng/mL (37%CV) at 6 h [24]. In contrast, Cmax values of 284 ng/mL and 63 ng/mL at 5 h were measured in this study. Furthermore, clindamycin has been reported to have a Cmax of 5.98 µg/mL (5.1% CV) approximately 1 h post-dose in patients with acute uncomplicated falciparum malaria; however, in this study, a Cmax of 4.6 µg/mL at the same time point was found [25]. Overall, the assay demonstrated reliable quantification of artesunate and dihydroartemisinin samples up to 8 h post-dose. Additionally, proguanil, cycloguanil, and pyronaridine samples could be measured reliably for up to 24 h after administration, with some samples remaining above the limit of quantification even at day 7 and beyond. Clindamycin samples were consistently quantifiable above the LLOQ for up to 12 h following the dose administration.

## Discussion

The present multiplex assay developed for the simultaneous quantification of artesunate, dihydroartemisinin, proguanil, cycloguanil, pyronaridine, and clindamycin has been successfully validated in accordance with the EMA Guideline for Bioanalytical Method Validation. This assay demonstrates robust analytical performance marked by high accuracy, sensitivity, and stability, suitable for extended analytical run times due to excellent autosampler stability.

The assay achieved LLOQs of 1 ng/mL for proguanil and 0.2 ng/mL for cycloguanil, surpassing previously reported LC–MS/MS benchmarks of 1.5 ng/mL and 0.5 ng/mL, respectively [13]. For the other analytes, while the LLOQs were comparable to those reported in existing LC–MS/MS methods, they did not exceed previously established lowest values of 0.39 ng/mL, 0.13 ng/mL, 1 ng/mL, and 1 ng/mL for artesunate, dihydroartemisinin, pyronaridine and clindamycin, respectively [10, 26, 27]. For both proguanil and clindamycin, the signal strength at the LLOQ suggests potential in reliably quantifying even lower concentrations. However, this possibility was not further explored due to the planned application of this assay in a clinical study, where it was anticipated that most samples would present with higher concentrations. The decision to maintain the calibration range within a 100-fold scope was deliberate and aimed at optimizing the assay’s precision and reliability.

Lindegardh et al*.* [28] reported that artemisinin-derivatives exhibit potential reactivity with hemoglobin and hemolytic compounds in the presence of organic solvents, leading to analytical challenges in the form of the degradation of the artemisinin-derivatives. Building on the insights from Lindegardh et al. [28], Hodel et al*.* [10] who demonstrated that certain sample preparation techniques can mitigate this degradation in haemolyzed plasma. Hence, the procedure from Hodel et al*.* [10] involving the evaporation of acetonitrile followed by reconstitution in a 50:50 mixture of aqueous buffer and methanol was implemented. The results demonstrated that this sample preparation does not lead to increased degradation in the presence of haemoglobin and haemolytic compounds, unlike conventional protein precipitation techniques. [10]. Although solid-phase extraction has also been shown to effectively address these stability challenges [8, 20], the method proposed by Hodel et al*.* [10] requires less equipment and fewer laboratory materials, providing a more economical option.

The dilution of samples in this study using method A deviated from the standard approach recommended by the EMA, which suggests diluting the sample with the matrix before sample preparation (method B). As the multitude of analytes present in the pharmacokinetic study had varying time points for their respective dilutions, a workflow was devised to avoid preparation of samples in both a diluted and non-diluted forms. The injection volume was decreased to effectively dilute the sample. While the analyte to I.S. ratio remained unchanged by the lower injection volume, the intensity of the analyte decreased, falling within the intensity range shown to be linear. It was also demonstrated with 15 replicates each that diluting the I.S. by 20-fold or 40-fold resulted in a linear decrease in response. Therefore, it was possible to achieve accurate and precise results while the ratio of analyte and I.S. was outside the calibration range. The method was tested and successfully validated. In the context of this assay, the method showed to be accurate and precise and showed to be a far more economical approach than the more common method B.

Long-term stability studies demonstrated that most analytes remain stable for up to 7 months, with many remaining stable for up to 12 months. Specifically, both artesunate and dihydroartemisinin have been reported to be stable for up to 12 months. Artesunate showed recoveries between 100 and 107%, while dihydroartemisinin showed recoveries of 85% and 88%, respectively [26]. The findings were consistent with these trends, though slightly higher degradation rates for dihydroartemisinin were observed, resulting in recoveries of less than 85%. Similarly, pyronaridine was reported to be stable for up to 12 months, aligning with the results in this study [11]. Previous studies have indicated that clindamycin exhibits minimal degradation even after 12 months of storage [29]. However, in this study, clindamycin showed recoveries slightly above 70% at both 7 and 12 months, contradicting the reported stability. Since no degradation was observed from 7 to 12 months, it is possible that an issue may have occurred during the baseline measurement, resulting in lower-than-expected recoveries. Long-term stability data for proguanil and cycloguanil have only been reported up to 3 weeks at − 80 °C [14]. It was found that cycloguanil exhibited excellent stability up to 12 months. However, the stability tests for proguanil showed inconsistent results, with a recovery of only 83% at 7 months and 95% at 12 months. No explanation was found to explain this discrepancy; however, it is likely due to an error in one of the measurements.

Despite its strengths, the assay faces inherent challenges. Initially, it was also attempted to include atovaquone into the assay. Yet, atovaquone does not ionize adequately with the electrospray ionization source used in this method, while proguanil presents difficulties under atmospheric pressure chemical ionization . Consequently, simultaneous mass spectrometric detection of these compounds is not feasible, creating the need for a separate assay for the analysis of atovaquone.

One limitation of the presented assay is the absence of deuterated internal standards. Although these standards are available, their high-cost poses a significant barrier to the development and utilization of an LC–MS/MS assay. Given the finding that trimipramine-d3 yielded excellent accuracy and precision with minimal matrix effects, it was decided to adopt this solution. This choice significantly reduces overall costs, facilitating easier implementation. Furthermore, recovery was not tested as it is not part of the EMA guidelines.

Regarding pyronaridine, significant carry-over was observed. This issue was addressed by implementing solvent injections following high-concentration samples. Although this successfully prevented interference of the carry-over at lower concentrations, it increased the overall run time and the need to sort samples according to their expected concentration before the run, making it infeasible to measure blinded samples containing pyronaridine. Blessborn et al*.* [11] reported a similar issue concerning the carry-over of pyronaridine, finding their carry-over to be significantly higher at 160%, compared to the presented assay, which exhibited a carry-over of 80%. To address this, the authors implemented a 4-step needle washing protocol between each injection. Recognizing that a memory effect on the column was likely the primary contributor to the carry-over, Schouten et al. [17] opted for a linear gradient elution instead of block gradients, successfully mitigating the issue. In the attempts to reduce carry-over, various needle washing solutions were tested, including different mixtures of 2-propanol and water, the addition of formic acid, and alternative solvents such as methanol and acetonitrile in place of 2-propanol. Unfortunately, none of these modifications resulted in a significant reduction in carry-over. Additionally, the system utilized in these experiments was not configured to accommodate multistep needle washing protocols. Altering the gradient would have either substantially increased the run times or compromised the retention and peak shapes of other analytes. Hence, it was determined that injecting three blank samples following each high-concentration pyronaridine sample represented the most economical and feasible solution to address carry-over while maintaining assay integrity.

A further limitation of this study is that pyronaridine was only measured in plasma, rather than in whole blood. Pyronaridine demonstrates a high distribution ratio between whole blood and plasma (1.6–9) [30], indicating that a substantial proportion of the drug is located within red blood cells. Consequently, whole blood is typically the preferred matrix for its analysis. Unfortunately, only plasma samples were available for this investigation, leading to the omission of whole blood as a matrix for analysis. Despite this limitation, measuring plasma concentration still provides valuable insights into the pharmacokinetics of pyronaridine.

In summary, this multiplex assay represents an advancement in the field of anti-malarial drug analysis, offering a comprehensive method for pharmacokinetic studies of these novel anti-malarial combination therapies.

## Conclusion

The developed multiplex assay provides a robust and efficient tool for the pharmacokinetic analysis of novel anti-malarial combination therapies, validated to EMA standards. It achieves high accuracy and sensitivity, with particularly low limits of quantification for proguanil and cycloguanil. While challenges such as the inability to simultaneously analyse proguanil and atovaquone remain, the assay effectively quantifies artesunate, dihydroartemisinin, pyronaridine, and clindamycin. Though pyronaridine carry-over requires careful sample management, the assay remains a valuable asset in advancing the understanding of these novel combination therapies.

## Data Availability

The authors declare that the data supporting the findings of this study are available within the paper. Should any raw data files be needed in another format they are available from the corresponding author upon reasonable request.

## Abbreviations

ACT: Artemisinin-based combination therapy
TACT: Triple artemisinin-based combination therapy
MDACT: Multi-drug antimalarial combination therapies
LC-MS/MS: Liquid chromatography - tandem mass spectrometry
HPLC: High performance liquid chromatography
LLOQ: Lower limit of quantification
I.S.: Trimipramine-d3
DMSO: Dimethyl sulfoxide
CERMEL: Centre de Recherches Médicales de Lambaréné
KCCR: Kumasi Centre for Collaborative Research
CS: Calibration standards
QC: Quality controls
EMA: European medicines agency
CV: Coefficient of variation
Cmax: Maximal plasma concentration
